# Development and characterization of poultry collagen-based hybrid hydrogels for bone regeneration

**DOI:** 10.1590/acb370302

**Published:** 2022-05-13

**Authors:** Francisco Fábio Pereira de Souza, Jesús Alberto Pérez-Guerrero, Maria Janaína Paula Gomes, Fábio Lima Cavalcante, Men de Sá Moreira de Souza, Igor Iuco Castro-Silva

**Affiliations:** 1 Fellow PhD degree. Universidade Federal do Ceará – Postgraduate Program in Biotechnology of Natural Resources – Fortaleza (CE), Brazil.; 2 MSc. Universidade Federal do Ceará – Postgraduate Program in Biotechnology – Sobral (CE), Brazil.; 3 Fellow PhD degree. Universidade Federal do Ceará – Postgraduate Program in Chemistry – Fortaleza (CE), Brazil.; 4 PhD. Embrapa Tropical Agroindustria – Fortaleza (CE), Brazil.; 5 PhD. Universidade Federal do Ceará – Postgraduate Program in Biotechnology – Sobral (CE), Brazil.

**Keywords:** Hydrogels, Collagen, Apatites, Materials Testing, Guided Tissue Regeneration

## Abstract

**Purpose:**

Poultry by-products can contribute as an innovative natural source for the development of composites based on polymers and minerals aiming at bone regeneration. The objective of this study was the physicochemical and biological characterization of collagen-based hydrogels crosslinked with ultraviolet (UV)-riboflavin.

**Methods:**

Pure hydrogels of 100% collagen (G1) or hybrid hydrogels, 90% collagen:10% apatite (G2), 90% collagen:10% nanokeratin (G3), and 90% collagen:5% apatite:5% nanokeratin (G4) were characterized by scanning electron microscope, Fourier-transform infrared spectroscopy, differential scanning calorimetry, swelling degree and quali-quantitative histological analysis. Ectopic implantation in subcutaneous tissue in mice at one, three and nine weeks allowed to assess the inflammation (neutrophils, lymphocytes, macrophages, and giant cells) and repair (neovascularization, and connective tissue) to determine biocompatibility and the integrity of biomaterials to score their biodegradability. Histomorphometry on critical size defects in rat calvaria at one and three months evaluated the percentage of bone, connective tissue, and biomaterials in all groups.

**Results:**

The hydrogels presented porous microstructure, water absorption and physicochemical characteristics compatible with their polymeric and/or mineral composition. All materials exhibited biocompatibility, biodegradability, and low osteoconductivity. G2 showed greater density of new bone and biomaterial than the G1, G3 and G4.

**Conclusions:**

The collagen-apatite group formulation suggests potential for development as osteopromoting membrane.

## Introduction

Bone grafts and membranes used in guided bone regeneration (GBR) are indicated in medical and dental practice for restoring compromised function and aesthetics due to bone defects^1^. Products applied to GBR must have physicochemical characteristics that mimic the injured tissue, in addition to being safe and predictable in preliminary pre-clinical tests^2^^,^ 3. Biocompatible biomaterials are considered non-irritating and non-immunogenic when in contact with biological systems^4^. A membrane barrier must have adequate stability time to prevent the infiltration of soft tissue in the bone defect area, balancing its biodegradation with its osteopromoting capacity^5^. For a bone implant, it is desirable to have osteoconductivity, supporting the adhesion, proliferation and differentiation of osteogenic cells in close contact with the biomaterial^6^.

The growth of translational scientific research, biotechnology industry and consumer market of GBR biomaterials around the world has motivated the development of new implantable devices aimed at improving the quality of life of patients^1^^,^ 5. The xenogeneic origin has been widely studied due to its high availability compared to autogenous or allogeneic natural products and its promising results in tissue repair^3^^,^ 7 ^,^ 8. The poultry industry can be a source of raw material, and this reality is even more sensitive in Brazil, the second country in the world with the largest production of chicken protein, around 13 million tons per year, equivalent to the sum of all national production of beef, pork, and fish^9^. Within the context of circular economy, by-products of skin, feathers and bones could return to the production chain for the synthesis of biomaterials^9^^-^ 11, thus converging with the global sustainable development goals^12^.

Type I collagen from chicken skin shows great similarity to the fibrous matrix of mammals, in which it represents a major protein, with cell-carrying capacity, binding effect and biocompatibility, despite weak interactions (H bonds, hydrophobic, and electrostatic interactions) associated with its biodegradability^13^^-^ 15. Poultry bioapatite has carbonate substitutions in its mineral structure, which increases its solubility, bioactivity and makes its hardness more similar to the inorganic portion of human bone than stoichiometric hydroxyapatite^13^^,^ 16. Feather β-keratin is formed by parallel or anti-parallel polypeptide chains with intermolecular H bonds between the NH and C = O groups, rich in the sulfur amino acid cysteine, which explains its stability, poor solubility, hemostatic action, and positive effect on skin wound repair^4^^,^ 10 ^,^ 17. Association of compositions such as collagen blends and other polymers (alginate, chitosan, and cellulose) could overcome the disadvantages of collagen alone, such as low mechanical and thermal resistance and accelerated enzymatic degradation^15^. Collagen-apatite scaffolds suggest greater predictability of regenerative potential associated with bone mimicry^3^^,^ 18 ^-^ 23. The use of organic and inorganic poultry matrices in hybrid form for GBR procedures would represent a technological innovation^13^. Furthermore, keratin in the organic phase together with hydroxyapatite may also represent an alternative composite for GBR^8^.

The biomaterials manufacturing process must predict their biological effectiveness and ease of use^1^. Hydrogels are three-dimensional polymeric networks capable of retaining large amounts of water and biological fluids, being classified into chemical (permanent, with covalent bonds) or physical (reversible, with inter or intramolecular interactions, H bonds or ionic bonds)^23^^,^ 24. The high hydrophilicity of hydrogels determined by amine, amide, carboxyl and hydroxyl groups simulates the extracellular matrix and promotes a favorable environment for cell infiltration, adhesion, proliferation, and differentiation^13^. The polymeric networks of hydrogels made up of polar chains are reinforced by chemical, thermal, ultraviolet (UV) or other polymeric cross-links, which makes it possible to modulate their dissolution^13^^,^ 14. Isolated crosslinking of collagen by UV irradiation using a photoinitiator such as riboflavin (water-soluble vitamin B2) is promising, as the reaction is fast, does not generate irritating by-products to the tissues and even sterilizes the material itself^23^^,^ 24. However, the synthesis and characterization of collagen-based hybrid hydrogels with UV-riboflavin crosslinking is still incipient, and its applicability in biological systems remains a knowledge gap that should be further investigated^13^^,^ 14 ^,^ 23 ^,^ 24.

The aim of this study was to evaluate the structure, biocompatibility, biodegradation and osteoconductivity of physical hydrogels crosslinked with UV-riboflavin based on collagen, apatite and nanokeratin derived from poultry industry waste for use in GBR procedures.

## Methods

### Ethical aspects

Access to biological material was previously registered in the National System for the Management of Genetic Heritage and Associated Traditional Knowledge (SisGen, Brazil), under registration number A576649.

This study adopted the international principles of Replacement, Reduction and Refinement in Animal Research: Reporting of In-vivo Experiments (3R-ARRIVE guidelines). The protocol was approved by the Ethics Committee on Animal Use of the Universidade Federal do Ceará (UFC-CEUA, Brazil), under registration number CEUA-UFC Sobral 04/17.

### Hydrogel processing

All fresh raw materials from *Gallus gallus domesticus* were derived from the broiler processing waste of a poultry industry in Fortaleza, Ceará, Brazil. Each component of the poultry hydrogels was obtained separately, following the protocol previously described by Souza et al.^11^.

Collagen was obtained from chicken skin after undergoing sanitization in sodium hypochlorite, removal of non-collagenous proteins with 0.05 mol/L sodium hydroxide 1:10 (w/v), lipids with 10% ethanol 1:10 (w/v), and minerals with 0.05 mol/L ethylenediamine tetraacetic acid (EDTA) 1:10 (w/v) at 8°C for 24 h, washing for neutralization, exposure to 0.5 mol/L acetic acid 1:25 (v/v) at 8°C for 72 h, filtration of non-soluble particles, storage in 0.9 mol/L sodium chloride for 24 h, redissolving in 0.5 mol/L acetic acid, centrifugation of the precipitate, dialysis against 0.02 mol/L sodium phosphate at 8°C for 48 h in 12-14 KDa membranes and against distilled water until neutral pH, freeze-drying and grinding.

Apatite was obtained from chicken foot bones using the alkaline hydrothermal method following immersion in 2% sodium chloride 1:10 (w/v), autoclaving, grinding, removal of lipids in acetone:ether solution 1:2 (w/v) and proteins with 4% sodium hydroxide 1:20 (w/v) for 24 h each, washings for neutralization, calcination at 500°C for 18 h, final wash and drying in an oven at 80°C.

Keratin was obtained from chicken feathers after sanitization with neutral detergent solution, removal of lipids with 70% ethanol, drying at room temperature, grinding, treatment with 5% sodium hydroxide (m/m) 1:40 (w/v) at 40°C under agitation for 4 h, filtration, dialysis against distilled water for 48 h, precipitation with 2 mol/L^-1^ hydrochloric acid until pH 4.2, centrifugation, washings for neutralization and freeze-drying.

To obtain the nanokeratin, there was solubilization in deionized water and addition of alcoholic solution of 8% glutaraldehyde under stirring for 24 h for the formation of nanoparticles, centrifugation, washes and ultrasonication cycles for complete removal of glutaraldehyde and, finally, freeze-drying.

Neutral physical hydrogels crosslinked with UV-riboflavin were prepared according to four formulations or experimental groups: G1 (100% collagen), G2 (90% collagen:10% apatite), G3 (90% collagen:10% nanokeratin), and G4 (90% collagen:5% apatite:5% nanokeratin). The preparation of hydrogels followed the protocol described by Heo et al.^24^ with adaptations.

Briefly, collagen was dissolved in 0.05 mol L^-1^ acetic acid, neutralized with 5% sodium hydroxide and buffered with 10-fold concentrated saline phosphate buffer in the proportion 1:9 (v/v). Then, apatite and/or nanokeratin were added at the desired concentrations and riboflavin 0.005% (m/v) under constant agitation until complete homogenization. The mixtures were placed in Petri dishes and exposed to UV radiation in a UV chamber (UV/Ozone ProCleaner Plus, Bioforce, United States of America) with a power of 15 mW/cm^2^ for 30 min to allow the crosslinking reaction. Finally, all formed hydrogels were lyophilized.

### Physicochemical characterization

The hydrogels were characterized by scanning electron microscopy (SEM), Fourier-transform infrared spectroscopy (FTIR) in attenuated total reflectance (atr) mode, differential scanning calorimetry (DSC), and swelling degree. For SEM, the samples were metallized with gold and analyzed in a Quanta 450 FEG environmental scanning microscope (FEI Company, United States of America) with a voltage acceleration of 15 kV at different magnifications. The FTIR analysis was performed in a FTLA 2000-102 device (ABB-Bomem Inc., Canada). DSC analysis was performed in DSC Q20 V24.9 Build 121 equipment (TA Instruments, United States of America) using nitrogen gas atmosphere with flow rate of 50 mL/min, heating rate of 1°C/min, equilibrium temperature of 25°C and final temperature of 90°C. For swelling degree, samples (10 mm × 10 mm) were weighed in time 0 (dry weight) and then immersed in distilled water, pH 7.1, at 25°C, and weighed during 11 time-intervals until 240 min (5, 10, 20, 40, 60, 80, 100, 120, 160, 200, and 240 minutes).

Afterwards, the membranes were removed from the water, and the excess water was removed using filter paper (Quanty; 8 μm) and weighed at the defined times. The swelling degree was expressed as the percentage of weight increase, compared to the dry weight, according to the Eq. 1:


$$D_{s}=\left(\frac{W_{s}-W_{d}}{W_{d}}\right)100\%$$ (1)

In which:

Ds = the swelling degree; Ws = the wet weight sample; Wd = the dry weight sample. All measurements were performed in quintuplicate.

### In vivo biological characterization

**Figure 1:**
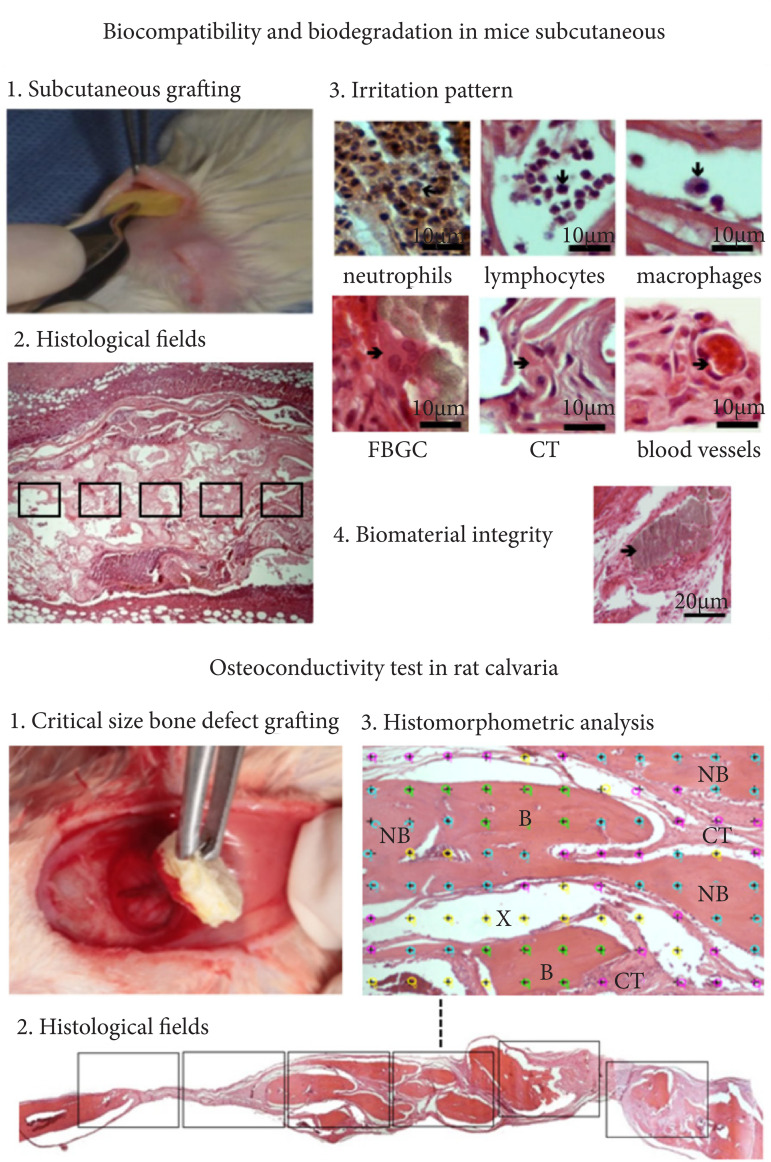
Steps of the procedure.

FBGC: foreign body giant cell; CT: connective tissue; B: biomaterial; NB: new bone; X: other structures.

The hydrogels were characterized for in-vivo biocompatibility, biodegradation and osteoconductivity. Biocompatibility and biodegradation analyses were performed in subcutaneous tissue of outbred Swiss mice (*Mus musculus*), males, young adults, weighing 30-40 g. Osteoconductivity analysis was performed in a critical size bone defect model in calvaria of outbred Wistar rats (*Rattus norvegicus*), male, young adults, weighing 220-250 g. During the experimental time, the animals were kept inside collective cages, according to their species and experimental group, in an acclimatized vivarium, with a 12-h light/dark cycle, pelleted feed and water *ad libitum*. All histological slides were examined under the supervision of an experienced pathologist. Figure 1 summarizes the in-vivo characterization procedures of this study.

### Ectopic subcutaneous implantation in mice

Forty-five animals were distributed according to different experimental conditions (five groups, three times, five specimens each). The animals were anesthetized intramuscularly with a 10% ketamine solution (Dopalen®, Sespo Indústria e Comércio LTDA, Brazil) at a dose of 100 mg/kg, and 2% xylazine (Anasedan®, Sespo Indústria e Comércio LTDA, Brazil) at a dose of 10 mg/kg. This was followed by trichotomy of the trunk-dorsal region and antisepsis with 0.5% aqueous chlorhexidine. A 1-cm linear incision was made, followed by tissue divulsion to form a subdermal pocket. Each animal received a subcutaneous implant of one of the hydrogels (G1, G2, G3 or G4) standardized with an area of 10 mm^2^ or remained without implant, only with a surgical bed filled with blood clot (C-). Then, the operated regions underwent simple sutures with 4-0 mononylon surgical thread measuring 0.2 mm in diameter.

At one, three and nine weeks after surgery, the animals were euthanized by an overdose of anesthetic solution, and immediate excisional necropsy of the area compatible with each surgery was performed, fixed in a 10% buffered formalin solution (v/v), pH 7, for 48 h. After fixation, necropsies were decalcified (in the case of groups G2 and G4) with an acidic rapid decalcifying solution (Allkimia, Brazil) for 12 h, washed in running water for 1 h, cleaved, dehydrated in increasing baths of 70 to 100% ethanol, bathed in xylene, impregnated and embedded in paraffin. Samples embedded in paraffin were microtomized in 4-μm thick sections and stained in hematoxylin-eosin (HE).

To characterize the biocompatibility and biodegradation, a qualitative and quantitative analysis was performed. Five images of each sample were captured in adjacent, non-overlapping fields in the center of the grafted or surgically manipulated area, spanning the whole length of each sample, in a camera Cybershot DSC-W300 Super Steady Shoot (Sony, Japan) coupled to the optical microscope FWL-1000 (Feldmann Wild Leitz, Brazil) using 40× objective lens and 4× digital zoom, making a final magnification of 1,600×.

For qualitative analysis, the slides of each experimental group were selected and morphologically described to represent the observed events. Quantitative analysis of biocompatibility or irritation pattern adopted the recommendations of ISO 10993-6^25^, considering as inflammatory criteria the presence of neutrophils, lymphocytes, macrophages, and foreign body giant cells, while as reparative criteria the presence of neovascularization and connective tissue. The standard evaluates such criteria in presence scores defined as 0 (absent), 1 (rare), 2 (moderate), 3 (intense) or 4 (overcrowding), generating a numerical system capable of determining the pattern of irritation. To arrive at the irritation pattern of each experimental condition (25 results, considering quintuplicates of animals and images of each test or control group), three equations were used: Eq. (2), for inflammation pattern equation (*I_x_*); Eq. (3), for repair pattern equation (*R_x_*); and Eq. (4), for total irritation pattern equation.


$$I_{x}=2\Sigma (Nt+L+M+FBCG)$$ (2)

In which:

*I_x_*: inflammation pattern by group; *x*: test or negative control group; *Nt*: mean score of neutrophils; *L*: mean score of lymphocytes; *M*: mean score of macrophages; *FBGC*: mean score of foreign body giant cells.


$$R_{x}=2\Sigma (Nv+CT)$$ (3)

In which:

*R_c_*: repair pattern by group; *x*: test or negative control group; *Nv*: mean score of neovascularization; *CT*: mean score of connective tissue.


$$IP_{x}=\left(I_{x}+R_{x}\right)-\left(I_{C-}+R_{C-}\right)$$ (4)

In which:

*IP_x_*: total irritation pattern by group; *x*: test group; *I_x_*: mean score of inflammation pattern by group; *R_x_*: mean score of repair pattern by group; *I_C–_*: mean score of inflammation pattern in negative control; *R_C–_*: mean score of repair pattern in negative control.

After the general calculations, the ISO 10993-6 standard adopts the negative control as the standard for the other groups, through subtraction. Therefore, their irritation pattern is scored as 0, and the experimental conditions follow the patterns: non-irritating (0-2.9), slightly irritating (3-8.9), moderately irritating (9-15), or severely irritating (> 15). The negative result is considered standard 0.

To analyze the biodegradation in the photomicrographs obtained in each test and control group, a similar pattern of scores based on ISO 10993-6 was built in parallel to grade the integrity or presence of the biomaterial by quartiles, defined as 0 (absent), 1 (minimum: up to 25%), 2 (mild: up to 50%), 3 (moderate: up to 75%) or 4 (predominant: above 75%).

The raw data were tabulated in Excel (Microsoft Office, United States of America), expressed graphically as a mean (± standard deviation) and statistically analyzed using the GraphPad Prism 7.0 software (GraphPad, United States of America), for intergroup comparisons according to criteria and experimental times described. For data with non-normal/non-parametric distribution, the Kruskal-Wallis’ test and Dunn’s post-test were applied, with the significance level of 5% (p < 0.05).

### Implantation in rat critical size calvarial defect

Sixty animals were distributed according to different experimental conditions (six groups, two times, five specimens each). The animals were anesthetized intramuscularly with a 10% ketamine solution (Dopalen®, Sespo Indústria e Comércio LTDA, Brazil) at a dose of 100 mg/kg and 2% xylazine (Anasedan®, Sespo Indústria e Comércio LTDA, Brazil) at a dose of 10 mg/kg. This was followed by trichotomy of the frontoparietal region of the skull and antisepsis with 0.5% aqueous chlorhexidine. A semilunar incision was made in the region, followed by a mucoperiosteal flap, elevated with the aid of a molt periosteum elevator, and the frontoparietal bone cortex of the skull was exposed.

In each animal, an 8-mm circular critical size defect was created using a trephine surgical drill (Sistema de Implantes Nacionais, Brazil) coupled to a counter-reducing angle with 20:1 rotation (Dentscler, Brazil) and a surgical micromotor (VK Driller Equipamentos Elétricos LTDA, Brazil) under continuous irrigation with cold and sterile 0.9% saline solution throughout the procedure. The osteotomized fragment was gently removed with the aid of an Ochsenbein #1 chisel.

The test groups had the bone defect filled with one of the test materials (G1, G2, G3 or G4). As a positive control (C+), the fragmented autogenous bone from the calvaria was used, and as a negative control (C-) the blood clot. Then, the operated regions underwent simple sutures with 4-0 mononylon surgical thread measuring 0.2 mm. It was applied with anti-inflammatory/analgesic medication Meloxicam (2 mg/kg, Ourofino, Brazil) subcutaneously every 12 h for two days.

At one and three months after surgery, the animals were euthanized by an overdose of anesthetic solution, and immediate excisional necropsy of the area compatible with each surgery was performed, fixed in a 10% buffered formalin solution (v/v), pH 7, for 48 h. After fixation, all necropsies were decalcified with an acidic rapid decalcifying solution (Allkimia, Brazil) for four days, washed in running water for 1 h, cleaved, dehydrated in increasing baths of 70 to 100% ethanol, bathed in xylene, impregnated and embedded in paraffin. Samples embedded in paraffin were microtomized in 4-μm thick sections and stained in HE.

To characterize the osteoconductivity, a qualitative and quantitative analysis was performed. Six images of each sample were captured in adjacent, non-overlapping fields, in a camera Cybershot DSC-W300 Super Steady Shoot (Sony, Japan) coupled to the optical microscope FWL-1000 (Feldmann Wild Leitz, Brazil) using 40× objective lens and 4× digital zoom, making a final magnification of 1,600×. For qualitative analysis, the slides of each experimental group were selected and morphologically described to represent the observed events. The following biological criteria were evaluated for the extent edge to edge of the bone defect, spanning its whole diameter: new bone, connective tissue and implanted material; and old (or native) bone, at the edges of the critical size defect, seeking to guarantee an unbiased and accurate analysis.

Histomorphometric analysis was performed using ImageJ 1.8.0 software (National Institutes of Health, United States of America), calibrated in micrometers/pixel. The biological criteria mentioned before were counted using a 130-point grid superimposed on each photomicrograph and from the absolute number of points obtained. The percentage density (%i) of each parameter was determined according to Eq. (5):


$$\%i=\left(\frac{pi}{P}\right)100\%$$ (5)

In which:

*p*_i_: the number of points of parameter *i*; *P*: the total number of points.

The raw data were tabulated in Excel (Microsoft Office, United States of America), expressed graphically as a mean (± standard deviation), and statistically analyzed using the GraphPad Prism 7.0 software (GraphPad, United States of America) for comparisons according to criteria and times experimental descriptions. For data with normal/parametric distribution, one-way analysis of variance (ANOVA) was applied. In the intergroup analyses, the Dunnett’s post-test was applied, comparing the difference between the means of the experimental groups in relation to the controls. In intragroup analyses, the unpaired Student’s t-test was applied to determine the differences between the means of each parameter of the same group at different experimental times. Significant differences were considered if p < 0.05.

## Results

### Structural presentation

**Figure 2:**
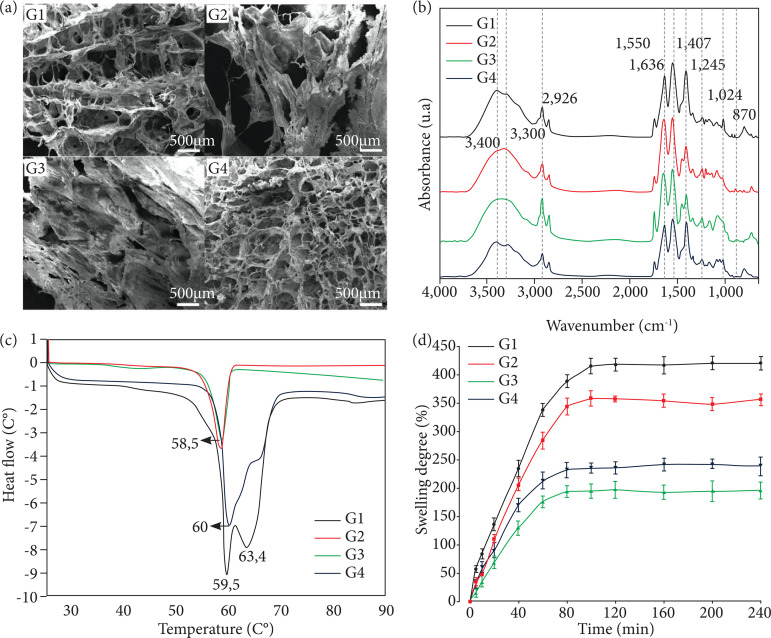
Physicochemical analysis of poultry collagen-based hydrogels: **(a)** scanning electron microscope; **(b)** Fourier-transform infrared spectroscopy; **(c)** differential scanning calorimetry; **(d)** swelling degree.

Figure 2a shows in SEM analysis that G1 and G4 presented filamentous and porous microstructure, with rounded and evenly distributed pores, with G1 exhibiting greater porosity and uniformity. G2 showed a filamentous microstructure of rough appearance, due to the presence of bioapatite granules, low porosity with large and irregular pores. G3 presented dense microstructure with laminar aspect and low porosity with elongated and irregular pores.

FTIR analysis shows in Fig. 2b for G1, G2, G3 and G4 bands of 3,400 and 2,926 cm^-1^ related to the symmetrical stretching of N-H bonds of amides A and B present in secondary structures of the ordered α-helix type of proteins, while the bands of 1,550 and 1,245 cm^-1^ are indicative of deformation of N-H bonds of amides II and III, respectively, confirming their collagen composition. FTIR curves demonstrate the presence of chemical groups characteristic of hydroxyapatites in G2 and G4, as indicated by the band of 1,407 cm^-1^, which corresponds to the presence of phosphate groups, and the bands of 1,024 and 870 cm^-1^, which correspond to the presence of carbonate groups and the replacement of phosphate groups by carbonate groups, respectively, the latter being typical of bioapatite. In addition, the band of 1,636 cm^-1^ in G3 and G4 corresponds to the stretching of C = O bonds of amide I carbonyl groups indicating presence of nanokeratin.

Figure 2c shows in DSC analysis the thermal behavior of hydrogels. Thermograms show the presence of subtle endothermic peaks and very similar between groups, ranging from 58 to 60°C. G1 and G4 presented a discrete endothermic peak at 37°C, which is related to the loss of moisture, and another more prominent one at 59.5 and 60°C, respectively, related to the breaking of chemical bonds and the beginning of the denaturation process. Similarly, G2 and G3 showed an endothermic peak at 58.5°C.

The materials that demonstrated higher and prolonged swelling capacities during the experimental time were G1 and G2, reaching an equilibrium after 100 min. Groups with composition of nanokeratin (G4 and G3) had lower water absorption and earlier saturation in swelling test, reaching an equilibrium after 80 min (Fig. 2d).

### Biocompatibility

**Figure 3:**
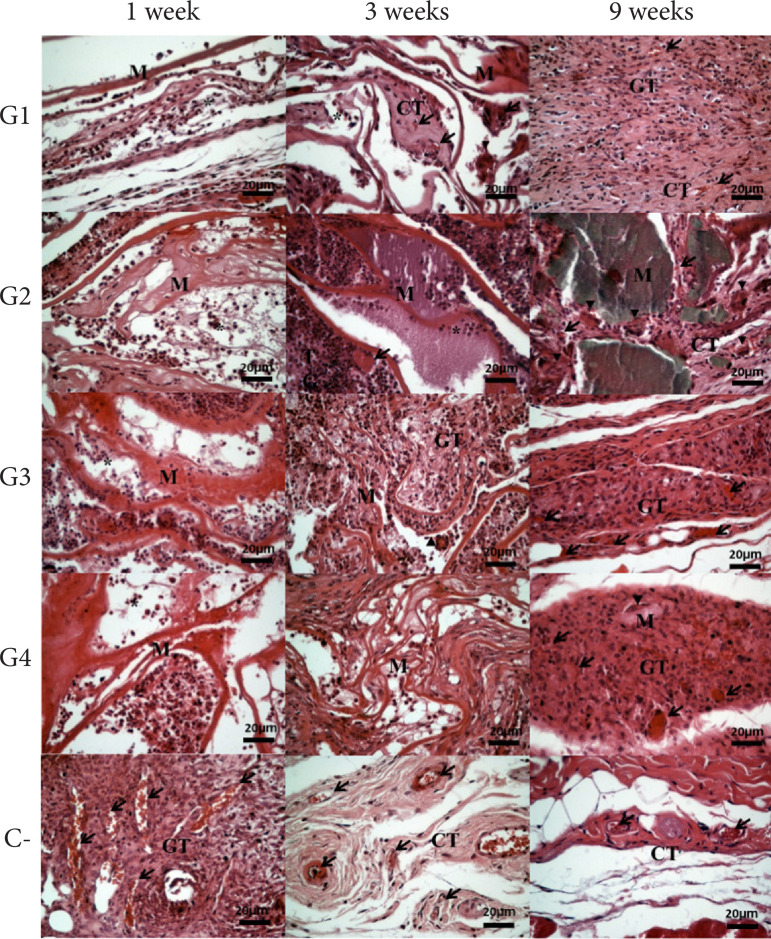
Biocompatibility analysis of poultry collagen-based hydrogels in subcutaneous tissue in mice at one, three and nine weeks.

Figure 3 demonstrates the qualitative biological response of implants in subcutaneous tissue. There was the presence of mixed inflammatory infiltrate, decreasing after one week, well vascularized granulation tissue and collagen deposition after three weeks. Additionally, G1, G2 and G4 had more prominent giant cells at three or nine weeks.

M: material; GT: granulation tissue; CT: connective tissue; *inflammatory cells; ▶ giant cells; →: blood vessels; staining: hematoxylin-eosin (HE).

**Figure 4:**
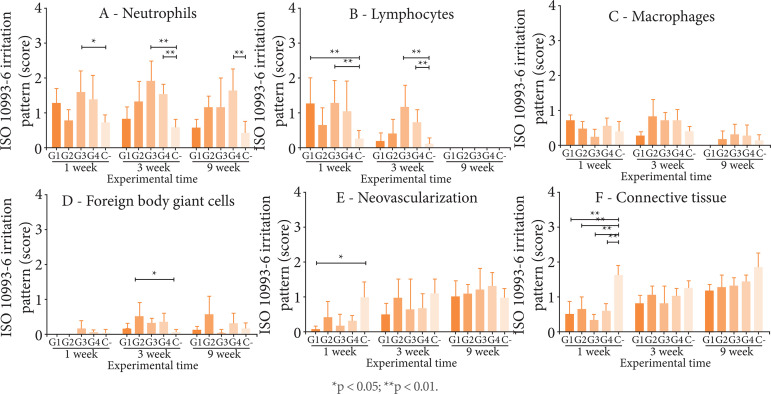
Inflammatory pattern and repair pattern of poultry collagen-based hydrogels in subcutaneous tissue in mice at one, three and nine weeks.

The quantification of the inflammatory and repair criteria is shown in Fig. 4. Presence of neutrophils was the highest for all treatments in one week, decreasing in three weeks and totally absent in nine weeks. Only G1 and G3 in one week (p < 0.001) and G3 and G4 in three weeks (p < 0.001 and p < 0.01, respectively) showed a significant increase in neutrophils when compared to C-. Similarly, presence of lymphocytes was higher between one and three weeks and decreased in nine weeks. Mean lymphocytes for G3 (p < 0.01) in one week, G3 (p < 0.001) and G4 (p < 0.001) in three weeks and G2 (p < 0.05) and G4 (p < 0.001) at nine weeks were superior to C-. Discrete presence of macrophages and presence of foreign body giant cells were also observed for all treatments higher than C- (p < 0.01) in three weeks.

*p < 0.05; **p < 0.01.

The irritation pattern induced by the grafted hydrogels ranged from non-irritating to mildly irritating (Table 1). It was observed that the pattern of irritation was more expressive for G3 and G4, which, as seen in the analysis of the inflammatory response, may be associated with the presence of nanokeratin.

Regarding the repair response, all treatments showed a gradual increase in the presence of neovascularization and connective tissue between one and nine weeks (Fig. 4). Mean connective tissue was lower than C- (p < 0.001) in one week and became equivalent after three weeks, which is probably related to the biodegradation of materials.

**Table 1:** Irritation pattern of poultry collagen-based hydrogels in subcutaneoustissue in mice at one, three and nine weeks.

Experimental times (weeks)	Experimental groups			
	G1	G2	G3	G4
One	1.64 (NI)	0.00 (NI)	1.68 (NI)	1.56 (NI)
Three	0.00 (NI)	3.56 (SI)	5.00 (SI)	3.72 (SI)
Nine	0.00 (NI)	1.88 (NI)	1.32 (NI)	3.00 (SI)

G1: 100% collagen; G2: 90% collagen:10% apatite, G3: 90% collagen:10% nanokeratin; G4: 90% collagen:5% apatite:5% nanokeratin; NI: non-irritating; SI: slightly irritating.

### Biodegradation

**Figure 5:**
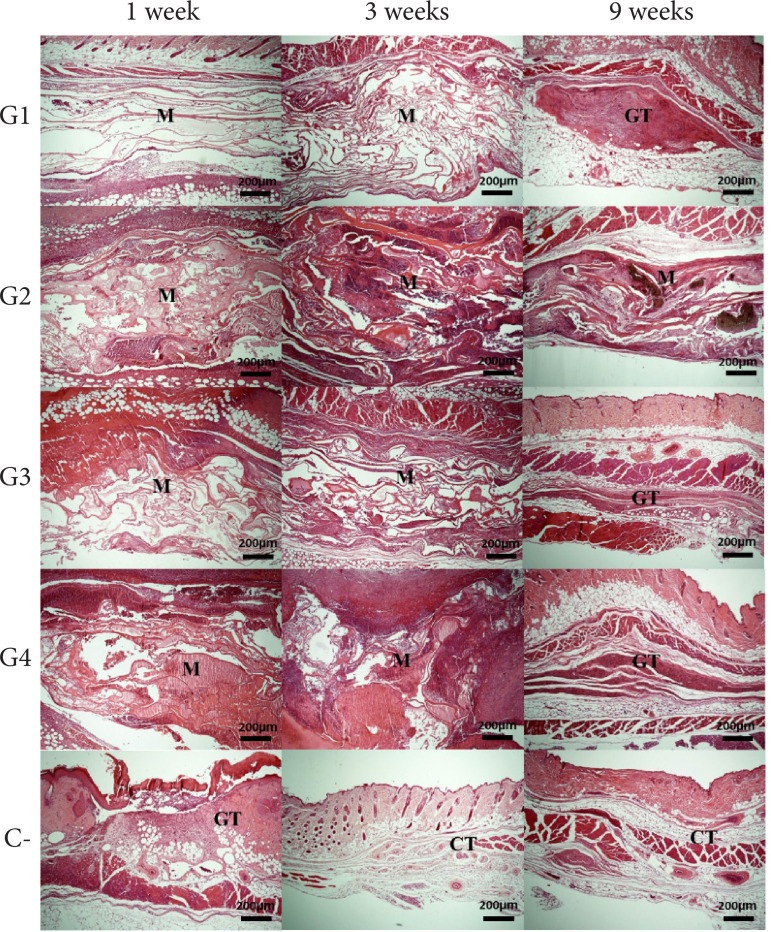
Biodegradability analysis of poultry collagen-based hydrogels in subcutaneous tissue in mice at one, three and nine weeks.

**Figure 6:**
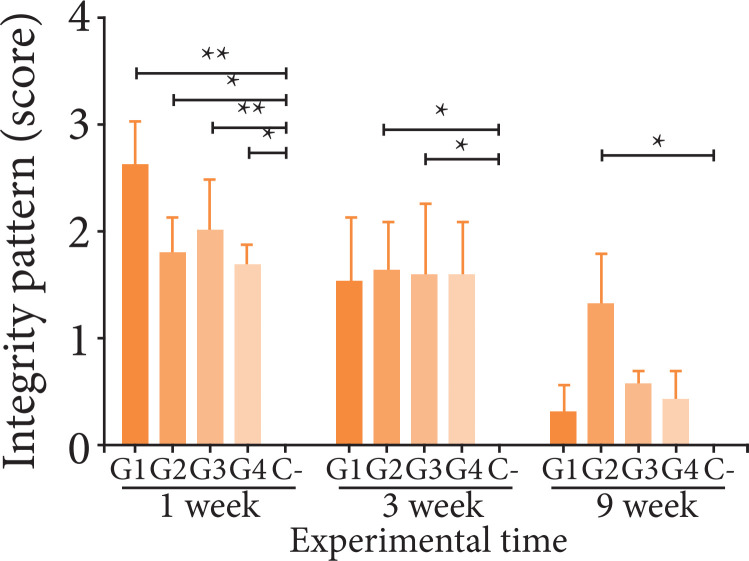
Integrity of poultry collagen-based hydrogels in subcutaneous tissue in mice at one, three and nine weeks.

Qualitative analysis of the presence of grafted hydrogels showed that G1, G3 and G4 underwent total resorption in up to three weeks, while G2 remained partially present in nine weeks, and no fibrous foreign body capsule was detectable circumscribing the area containing fragments of the tested materials (Fig. 5). Figure 6 shows the results of the quantitative analysis of material integrity, in which groups G1, G3 and G4 suffered a significant reduction between one and nine weeks (p < 0.001). Only G2 was still present at the end of nine weeks, which is probably related to the presence of bioapatite in a higher percentage in its composition.

M: material; GT: granulation tissue; CT: connective tissue; staining: hematoxylin-eosin (HE).

*p < 0.05; **p < 0.01.

### Osteoconductivity

**Figure 7:**
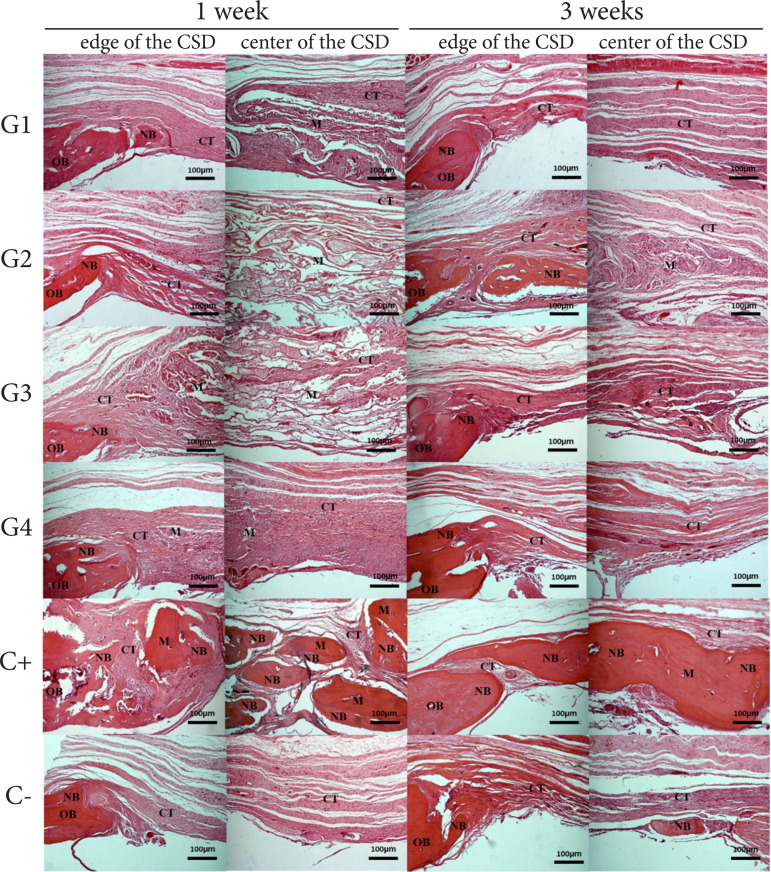
Osteoconductivity analysis of poultry collagen-based hydrogels in critical size bone defect in rat calvaria at one and three months.

CSD: critical size defect; NB: new bone; OB: old bone; M: material; CT: connective tissue; staining: hematoxylin-eosin.

Histological analysis of critical size defect in rat calvaria grafted with different hydrogels showed distinct profiles according to treatment and time (Fig. 7). All treatments presented newly formed bone adjacent to or in islands close to the defect border at one and three months. Regarding integrity, G1, G3 and G4 presented volume reduction in one month and complete resorption in three months, giving way to dense connective tissue. In contrast, G2 remained partially intact and permeated by dense connective tissue for up to three months. The C- also showed areas of newly formed bone in the center of the defect and abundant presence of connective tissue at one and three months. As for the C+, exuberant areas of newly formed bone were observed in the center of the defect at one and three months, with no qualitative change observed in the presence of connective tissue. Considering the histomorphometric data, the materials showed low osteoconductivity, as they underwent total resorption within three months, not preventing the infiltration of soft tissue into the bone defect area at this later time.

**Figure 8:**
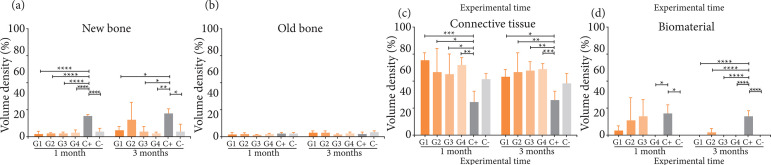
Percentage of new and old bone, connective tissue, and biomaterial of poultry collagen-based hydrogels in critical size bone defect in rat calvaria at one and three months.

The distribution of percentage of the criteria analyzed by histomorphometry according to treatment and time is shown in Fig. 8. Newly formed bone for all treatments ranged from 2.6 to 4% in one month, similarly to C- (5%), but lower than the C+ (p < 0.0001), which presented a percentage of 19.3%. In three months, there was a slight increase in the percentage of bone neoformation for G1, G3 and G4 (ranging between 2.8 and 6.5%), and a more expressive increase for G2, which presented 15.7%, a percentage statistically equivalent to C+ (21.8%). The percentage of old bone did not vary between the test and control groups. The percentage of connective tissue for all treatments ranged from 56.6 to 69.4% in one month and from 53.6 to 61.8% in three months, being higher than C+ (30.8 and 32.1%, respectively) (p < 0.001). Regarding the integrity of hydrogels grafted in critically sized bone defects, only G2 remained present at the end of three months, although with a percentage (2.1%) much lower than C+ (17.5%) (p < 0.001).

*p < 0.05; **p < 0.01; ***p < 0.001; ****p < 0.0001.

## Discussion

Porosity is an important feature, as it facilitates cell-biomaterial interaction and cell proliferation^26^^-^ 28. In the case of hydrogels, the porous microstructure can favor cell proliferation and differentiation and, consequently, the formation of new tissues, including connective and vascular tissues^4^^,^ 29 ^-^ 31. Thus, it is believed that the three-dimensional and porous structure of the hydrogels evaluated in this study favored the proliferation of fibroblasts and endothelial cells, thus contributing to a gradual increase in collagen deposition and formation of new blood vessels, as observed in in-vivo assays.

FTIR analysis showed spectra with similar profiles between implants with the presence of chemical groups with characteristic bands of protein structures, such as collagen and nanokeratin^11^^,^ 15 ^,^ 17 ^,^ 21 ^,^ 23, and minerals, such as bioapatite^11^^,^ 16 ^,^ 18 ^,^ 21 ^,^ 23, as shown in the literature. The high similarity between the thermal behavior of the materials is directly associated with the majority presence of poultry collagen in the formulations^11^^,^ 13.

The greater swelling degree or ability of the biomaterial in interact with the aqueous solvent could be related to the chemical composition itself and crosslinking process^5^^,^ 13 ^,^ 14. The results corroborated the literature due to greater hydrophilicity of collagen^15^ and apatite^16^ in relation to keratin^17^. Collagen-based hydrogels crosslinked with UV-riboflavin in this study showed more continuous swelling than oxidized cellulose membranes in which aldehyde is released in the reaction that induces indirect low crosslinking and, therefore, presents a higher initial water absorption^5^, reinforcing the importance of processing for understanding the behavior of biomaterials. The swelling can accelerate the degradation due to hydrolysis of biomaterials, in addition to local enzymatic and cellular action^5^^,^ 13 ^,^ 14. Therefore, it was necessary to compare the in-vivo integrity of the hybrid hydrogels tested.

By contextualizing the polymeric and ceramic derivation of grafted hydrogels, the biological response found converges with the biocompatibility standard recommended by ISO 10993-6^25^ and literature. Studies in murine subcutaneous tissue demonstrated mild to severe inflammation within one week, moderate within three weeks, and mild to absent within nine weeks, with a gradual increase in collagenization and neovascularization from one week onwards in xenogeneic collagen membranes^32^^,^ 33, hydrogels of poultry keratin^4^^,^ 34 ^,^ 35, ceramic implants^3^^,^ 36 ^,^ 37, alginate^38^ or fibroin-based materials^39^. The increased number of neutrophils in one week is compatible with the acute inflammation, being the predominant cell type^40^^,^ 41. In addition, the decrease in lymphocytes after three weeks and the discrete presence of macrophages and giant cells are indicative of a moderate inflammatory response or ending of the inflammatory process^42^^,^ 43. Interestingly, a more pronounced inflammatory response was observed for groups G3 and G4, which contained nanokeratin in their composition. It is believed that the inflammatory profile is related to the presence of nanokeratin, since the literature points out to accentuated inflammatory responses to polymeric materials^38^^,^ 40, including keratin extracted from chicken feathers^4^^,^ 11 ^,^ 13.

Thematic studies corroborate these results and show collagen-based biomaterials grafted in murine subcutaneous tissue that underwent total resorption between three and nine weeks post-grafting^30^^,^ 33 ^,^ 38 ^,^ 42. On the other hand, ceramic materials, such as biological apatites, show greater integrity over time, revealing a partial biodegradation profile between three and nine weeks post-grafting^29^^,^ 35 ^,^ 44 ^,^ 45. Composites containing hydroxyapatite at different concentrations exhibit moderate to slow biodegradation over time, with partial resorption between four and 10 weeks, as observed in composites of collagen and hydroxyapatite (25:75 ratio)^3^, collagen and nanohydroxyapatite in different proportions^22^ and hydroxyapatite and fibroin^39^.

Biodegradation of materials occurs through the action of inflammatory infiltrate cells that fragment and phagocytose the product, being a very important process, as it favors tissue regeneration in the area previously occupied by the implant^2^^,^ 40 ^-^ 42. The fibrous architecture and crosslinking of biomaterials from different xenogeneic origins can explain different rates of biodegradation, as observed in an assay with enzymatic action of trypsin and collagenase in up to 50 days, in which equine collagen products exhibited greater biodegradation than swine and bovine^46^.

The results observed for bone neoformation of all treatments at one and three months converge with results from thematic studies using critical size defect model in rat calvaria. Polymers such as collagen, poly(lactic-co-glycolic acid) or demineralized bone matrices induced 3 to 10% bone neoformation between two and 12 weeks^20^^,^ 46 ^,^ 47. For ceramic materials (HA, β-TCP, β-TCMP) or bioglasses, of different formulations, the percentage of bone neoformation shows a wide variation, from 1.8 to 25% between two to 12 weeks post-implantation^48^^-^ 50. As for composites with collagen and HA, β-TCP or β-TCMP, the literature shows variation between 3.4 and 9% of bone neoformation between two and 24 weeks^3^^,^ 7 ^,^ 20 ^,^ 51 ^,^ 52.

The high percentage of connective tissue observed for the treatments may be directly related to the partial or total resorption of materials within three months. Literature shows that collagen-based composites associated with hydroxyapatite, β-TCP, β-TCMP when applied to critical size defects showed a lower percentage of connective tissue ranging between 17 and 44% in one month^7^^,^ 46 ^,^ 51 ^,^ 52. These results suggest evidence of synergism between collagen and apatites, in which the mineral component would be associated with the control of connective tissue invasion in the critical defect area.

The higher percentage of mineral component of G2 (10% apatite) could be directly related to the lower rate of material resorption in comparison to the other groups, as shown by a study with a composite of keratin and hydroxyapatite that remained partially stable up to three months after implant^8^. Such evidence supports the influence of the mineral component on the resorption rate of polymer-based composites. Although resorption is a very important feature when the development of new grafts is intended, ideally it occurs within a period of time that is compatible with the target tissue repair time^46^^,^ 52. In the case of developing new bone substitutes, the ideal resorption time must be compatible with the time required for bone formation or a minimum of one month for use as a membrane barrier^5^^,^ 8. Such findings indicate that the materials have low potential for applicability as bone substitutes, however they suggest their potential use as osteopromoting membranes in guided bone regeneration procedures^1^^,^ 46.

## Conclusions

The poultry collagen-based hybrid hydrogels with UV-riboflavin crosslinking presented physicochemical characteristics compatible with their polymeric and/or mineral composition and porous microstructure.

All materials exhibited biocompatibility, biodegradability and low osteoconductivity. However, the collagen-apatite group showed greater functional stability than the collagen, collagen-nanokeratin or collagen-apatite-nanokeratin formulations suggesting potential for development as an osteopromoting membrane.
